# Mobile Clinical Decision Support System for the Management of Diabetic Patients With Kidney Complications in UK Primary Care Settings: Mixed Methods Feasibility Study

**DOI:** 10.2196/19650

**Published:** 2020-11-18

**Authors:** Hala Ibrahim Alhodaib, Christina Antza, Joht Singh Chandan, Wasim Hanif, Sailesh Sankaranarayanan, Sunjay Paul, Paul Sutcliffe, Krishnarajah Nirantharakumar

**Affiliations:** 1 Department of Community Health Sciences College of Applied Medical Sciences King Saud University Riyadh Saudi Arabia; 2 Division of Health Sciences Warwick Medical School University of Warwick Coventry United Kingdom; 3 Institute of Metabolism and Systems Research University of Birmingham Birmingham United Kingdom; 4 Institute of Applied Health Research University of Birmingham Birmingham United Kingdom; 5 Diabetes Centre University Hospitals Birmingham Birmingham United Kingdom; 6 Warwickshire Institute for the Study of Diabetes, Endocrinology and Metabolism Centre University Hospitals Coventry and Warwickshire Coventry United Kingdom; 7 The Royal Wolverhampton NHS Trust Wolverhampton United Kingdom; 8 Health Data Research UK Birmingham United Kingdom

**Keywords:** eHealth, clinical decision support application, diabetes mellitus, chronic kidney disease, feasibility study

## Abstract

**Background:**

Attempts to utilize eHealth in diabetes mellitus (DM) management have shown promising outcomes, mostly targeted at patients; however, few solutions have been designed for health care providers.

**Objective:**

The purpose of this study was to conduct a feasibility project developing and evaluating a mobile clinical decision support system (CDSS) tool exclusively for health care providers to manage chronic kidney disease (CKD) in patients with DM.

**Methods:**

The design process was based on the 3 key stages of the user-centered design framework. First, an exploratory qualitative study collected the experiences and views of DM specialist nurses regarding the use of mobile apps in clinical practice. Second, a CDSS tool was developed for the management of patients with DM and CKD. Finally, a randomized controlled trial examined the acceptability and impact of the tool.

**Results:**

We interviewed 15 DM specialist nurses. DM specialist nurses were not currently using eHealth solutions in their clinical practice, while most nurses were not even aware of existing medical apps. However, they appreciated the potential benefits that apps may bring to their clinical practice. Taking into consideration the needs and preferences of end users, a new mobile CDSS app, “Diabetes & CKD,” was developed based on guidelines. We recruited 39 junior foundation year 1 doctors (44% male) to evaluate the app. Of them, 44% (17/39) were allocated to the intervention group, and 56% (22/39) were allocated to the control group. There was no significant difference in scores (maximum score=13) assessing the management decisions between the app and paper-based version of the app’s algorithm (intervention group: mean 7.24 points, SD 2.46 points; control group: mean 7.39, SD 2.56; t_37_=–0.19, *P*=.85). However, 82% (14/17) of the participants were satisfied with using the app.

**Conclusions:**

The findings will guide the design of future CDSS apps for the management of DM, aiming to help health care providers with a personalized approach depending on patients’ comorbidities, specifically CKD, in accordance with guidelines.

## Introduction

Diabetes mellitus (DM) is one of the most common chronic diseases worldwide. The World Health Organization estimated that 422 million adults worldwide had diabetes in 2014 and 1.5 million died from it in 2012, while DM became the 7th leading cause of death in 2016 [1]. In the United Kingdom, the DM prevalence is estimated to rise to 5 million by 2025 [2]. The economic burden of the disease is equally high, with most of the costs associated with its complications [3]. Specifically, type 2 DM with chronic kidney disease (CKD) costs 49% more annually than type 2 DM without CKD [4]. Most of the complications of DM can be avoided with regular monitoring and good management [5,6]. Patient and health care education are important in diabetes care. Hence, a promising step is to consider using information technology in diabetes management, such as mobile apps and other eHealth solutions [7-9].

Advances in technologies may help understand and implement current guidelines more quickly. Doctors spend nearly 64% of their online time searching for information to support clinical decisions. The use of mobile clinical decision support system (CDSS) devices allows health providers to make rapid and more accurate decisions [8,10-12]. The World Health Organization recognizes that an increasing proportion of the population uses mobile health apps. As a result, the need for evidence-based guidance on the use of mobile health is required to advance integrated person-centered health services [13]. Attempts to use eHealth for diabetes management have been reported going back to the late 1970s and show promising outcomes [14-16]. More than 1100 apps relating to DM had been reported as of 2015 [17], but only 7%-8% of them were provider-directed [18,19].

Limited studies have evaluated CDSS app use in diabetes care [20-22], while the number of studies documenting implementation and evaluation is even lower [23]. Moreover, the existing literature has weaknesses in the quality of reporting methodological domains, cost-effectiveness of the apps, care providers’ assessment, and adverse effects of mobile intervention in clinical practice.

Based on the limitations of the existing literature, we conducted a feasibility study aimed at developing and evaluating a mobile app developed exclusively for health care providers to manage DM and CKD. To our knowledge, this is the first published feasibility study developing a provider-directed CDSS app specifically for management of patients with DM and comorbidities, such as CKD. The developed mobile app was evaluated in a controlled setting for its usability and impact on workflow and adherence to clinical guidelines.

## Methods

### Ethics Approval and Ethical Considerations

The University of Warwick’s Biomedical and Scientific Research Ethics Sub-Committee approved all stages of the study (RFGO-2014-786). Nurses were fully informed about the study and were given the participant information leaflet and participant consent form. Ethics approval and ethical considerations are described in detail at Multimedia Appendix 1.

### Research Framework

This study is based on the 3 key stages of the user-centered design framework, which is a generic, multidisciplinary, and user-oriented approach to software development, putting the intended users, their needs, and their requirements at the center [24].

### First Step: Requirements Gathering

#### Research Design and Setting

The first part of the study consisted of an exploratory qualitative study using face-to-face semistructured interviews with DM specialist nurses from hospitals and community health centers across West Midlands, United Kingdom. Diabetes Specialist Nurses who worked at local National Health Service (NHS) facilities and had a minimum of 2 years’ experience working with people with DM were eligible for the study.

#### Recruitment and Interview Process

The recruitment was performed mainly via emails or word-of-mouth. Once eligibility and consent were confirmed, an interview was scheduled. Interviews lasted 15-30 minutes and took place in a meeting room or office at the hospital or practice where the nurses worked. The interview topic was the use of mobile CDSS apps that assist nurses in managing aspects of diabetes. All interviews were recorded using a digital recorder. The recruitment, choice of interview type (face-to-face, semistructured), and further details are provided in Multimedia Appendix 2.

#### Sample Size and Qualitative Data Synthesis

A minimum sample size estimate of 10 DM specialist nurses was chosen, with continued sampling until the saturation point [7,25,26]. Saturation was reached after 15 interviews at which time the recruitment was stopped.

Thematic analysis was used, and interview recordings were transcribed verbatim by the researcher [9,27]. The computer-assisted qualitative data analysis software NVivo (version 10) was used to assist in the analysis process. In an attempt to minimize bias in the interpretation of the data, an experienced qualitative researcher was consulted on the conduct and analysis of this research.

### Second Step: Design and Development of the Mobile, Clinical Decision Support App

#### Requirements

The nurses’ feedback at the “requirements gathering” stage, other requirements gathered from the literature, and requirements from feedback and suggestions from 2 diabetes and endocrinology consultants were considered during step 2. The total requirements were divided into 3 categories: functional, technical, and medical. These are analyzed further in Multimedia Appendix 3.

#### Design and Development

The app was built by a software developer (Medic Genie), and the development process included two parts: design and coding. Both parts were done by visual programming, while a junior doctor provided well-defined guidance and verification of the correctness of the management pathway. Development of the app involved generating management pathways using the most recent National Institute for Health and Care Excellence (NG28) guidelines on DM, CKD, and hypertension. The guidelines used in the development of the management pathways, developed decision algorithm, and table of dose adjustments for CKD are provided in Multimedia Appendix 4, Multimedia Appendix 5, and Multimedia Appendix 6, respectively.

### Third Step: Evaluation Stage

#### Research Design

This component used multiple methods and quantitative and qualitative designs. The 3 main methods used for this part of the study included a randomized controlled experiment, usability testing, and a satisfaction questionnaire (Multimedia Appendix 7), with an aim of demonstrating the impact and acceptability of the app.

#### Recruitment

Two types of end users were considered as participants: junior doctors and DM specialist nurses.

The junior doctors were recruited for the randomized controlled experiment from the University Hospitals Birmingham NHS Foundation Trust as part of a teaching session on DM and renal complications. At the teaching session, 39 doctors were recruited through convenience sampling. During the piloting phase, junior doctors were randomly divided into 2 groups using software-generated random numbers. The intervention group had access to the developed app “Diabetes & CKD,” while the control group had access to paper-based guideline algorithms that informed the app’s development. At this stage, 2 case scenarios were prepared by a diabetes and endocrinology consultant from the University Hospitals Birmingham NHS Foundation Trust. The evaluation was conducted to assess how the app could support health care providers in terms of (1) workflow efficiency (measured by time to complete the tasks) and (2) adherence to clinical guidelines (measured by accuracy of the decision made, compared to the use of paper-based guideline algorithms). Those in the control group had access to paper-based guideline algorithms that informed the app development, while participants in the intervention group were given a link to the app. Both groups were asked to deal with 2 simulation-based case scenarios (Multimedia Appendix 8). Decisions made in each group were written on the provided answer sheets. At the end, intervention participants were asked to complete the satisfaction questionnaire (Multimedia Appendix 9).

The DM specialist nurses from the interview study who expressed interest in taking part were included. Although 15 DM specialist nurses were invited via email to take part in the usability testing session, only 3 were recruited at the Sandwell & West Birmingham Hospitals NHS Trust, United Kingdom. During the testing phase, nurses were asked to perform tasks using the case scenarios and to verbalize what they are doing while they were doing it. Broad questions were used to explore participants’ views and opinions during the session.

### Statistical Analysis

The answers for both groups in the randomized controlled experiment were blinded and scored against a model answer prepared in advance by the consultant. In the scoring scale, minor and major decisions were not scored equally; the weight varied. The scoring was carried out by an independent clinician not directly involved in the preparations of the case scenarios. Scores were compared and analyzed using independent samples *t* tests or Mann-Whitney U tests for hypothesis testing, as appropriate. Statistical analysis was carried out using SPSS software (version 20.0). For the satisfaction questionnaire, basic analysis was undertaken. Responses were read carefully several times; then, major patterns and trends were identified in the responses and summarized. The usability testing session was audiotaped and transcribed verbatim by a professional transcription company and analyzed using a narrative synthesis approach.

## Results

### First Step: Requirements Gathering

#### Participant Characteristics

Interviews were conducted with 15 DM specialist nurses from 4 hospitals and 2 community health centers across the West Midlands regarding the use of the mobile CDSS app. All were female, with an average age of 45 years and an average duration as a nurse specializing in DM of 10 years. The participants’ characteristics are summarized in Multimedia Appendix 10. They all were owners of a tablet or smartphone. Moreover, 14 of 15 (93%) used a device provided by the Trust during clinical practice.

#### Interview Findings

**Figure 1:**
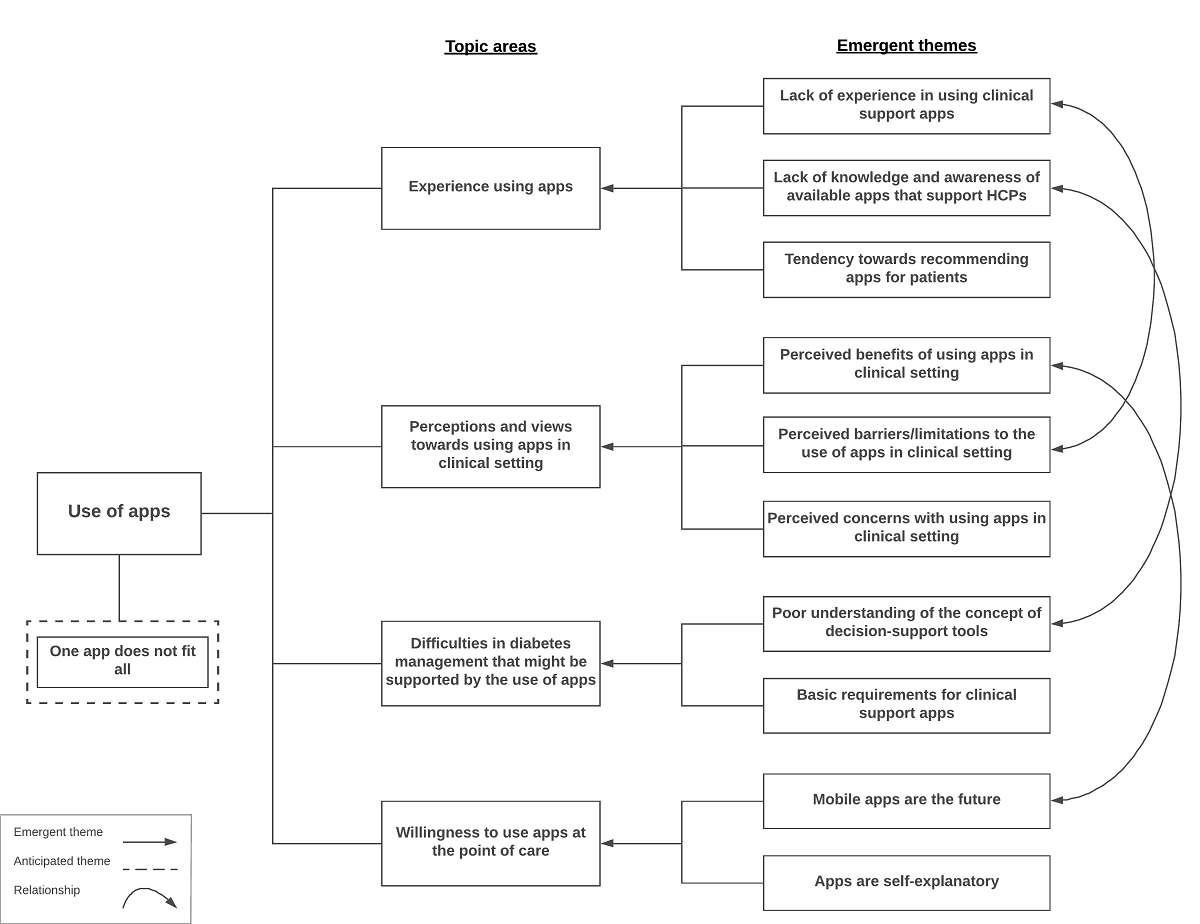
Themes identified during the interviews in the "requirements gathering" stage and their relationships with each other. HCP: health care professional.

The interviews with nurses identified varying themes related to 5 main areas: prior experience with using apps, perceptions and views of using apps in clinical practice, challenges in DM management, willingness to use apps, and one app does not fit all. The “one app does not fit all” area emerged during the interviews but did not fit under any of the 4 main investigated areas. People have different needs and preferences, range of skills, and degree of motivation, so one app will not fit all, as believed by most nurses. The themes identified by the interview and their relationships are shown in Figure 1. Examples of the DM specialist nurses’ answers that helped formulate the conclusions are provided in Multimedia Appendix 11.

Generally, nurses urged for apps that are simple, short, and to the point. Clinical support apps need to work across multiple mobile platforms, not require a WiFi connection, be visual, be interactive, and not require the inputting of too many details. Finally, they agreed that clinical apps need to be customized locally. Overall, nurses expressed a strong willingness to use apps in clinical practice.

### Second Step: Design and Development of the Mobile, Clinical Decision Support App

**Figure 2:**
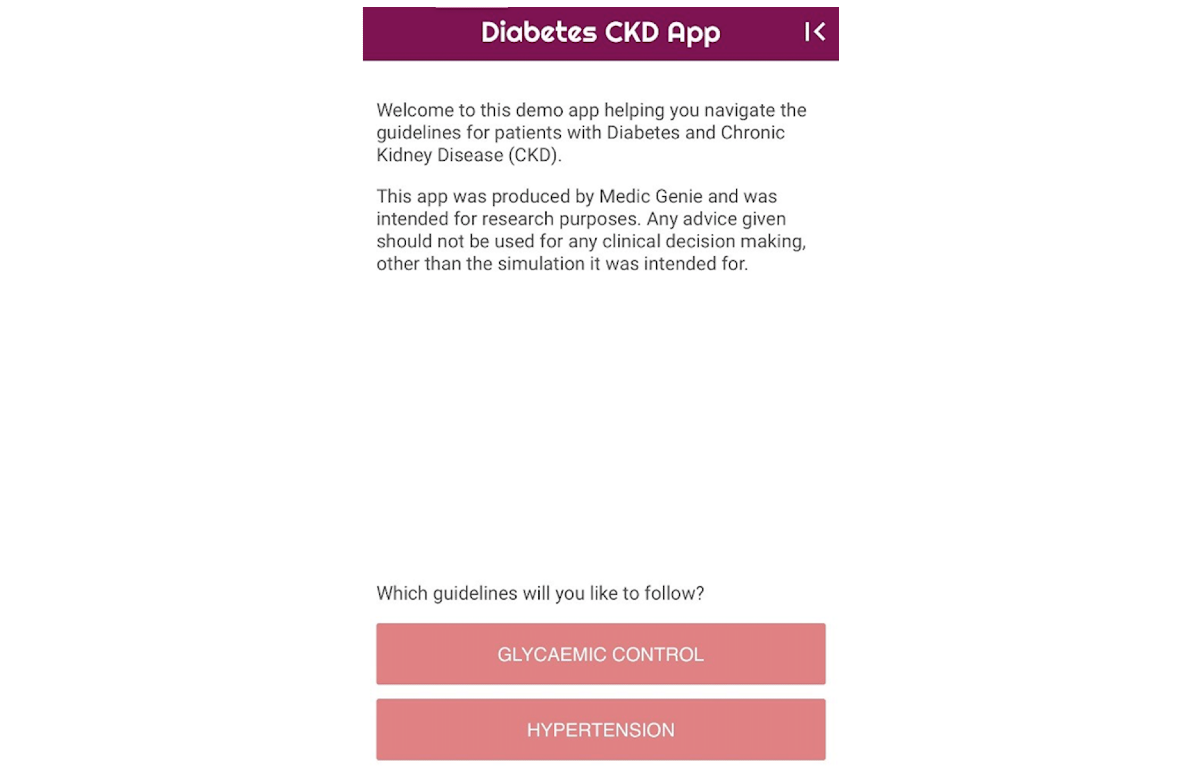
Home screen of “Diabetes and CKD” app.

**Figure 3:**
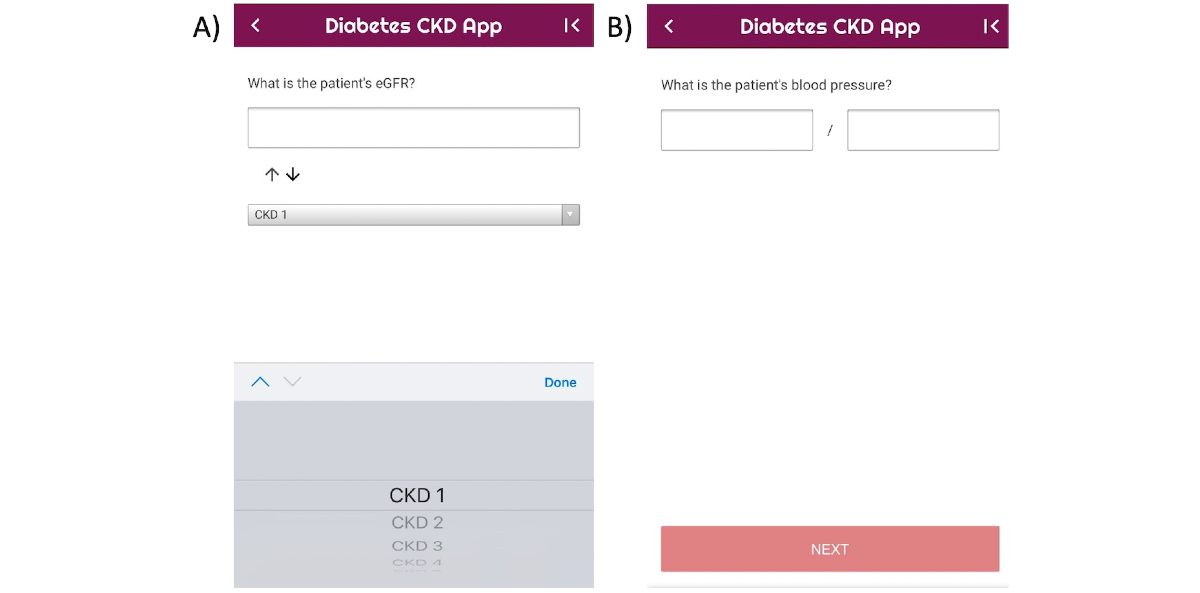
Examples of data entry screens.

**Figure 4:**
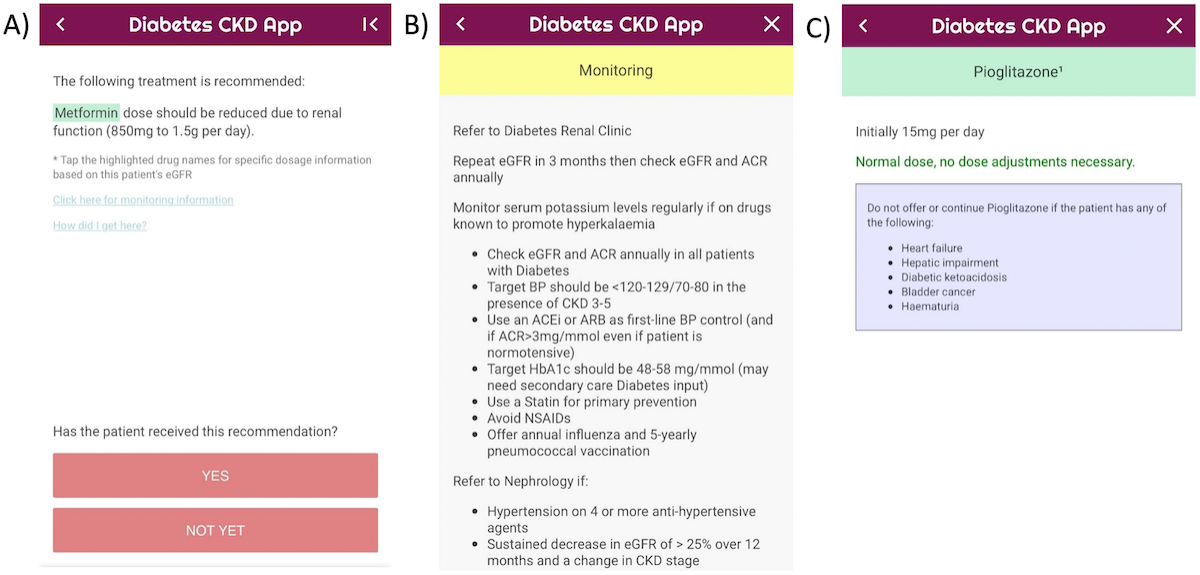
Examples of recommendation screens.

At this stage, the “Diabetes & CKD” mobile CDSS app for the management of adult patients (≥18 years old) with type 2 DM and CKD was designed and built [28]. The aim of the functionality of the app is to work out a personalized treatment plan based on patient’s parameters. The app provides an easy-to-follow interface. When possible, dropdown menus, predefined lists, or checkboxes were considered in an attempt to reduce the amount of typing required for data input. Error checks for numerical variables were applied to ensure that the inputted value was in range. The app consists of 3 main types of screens: home screen (Figure 2), data entry screens (Figure 3), and recommendation screen (Figure 4). The home screen welcomes the user to the app and guides the user to 2 possible choices: follow either the glycemic control guidelines or the hypertension guidelines. At the next screen, patient’s personalized data are inputted by the health provider, and the app provides recommendations based on guidelines to treat the patient accordingly.

### Third Step: Evaluation

#### Participant Characteristics

In the controlled experiment, 39 junior foundation year 1 doctors were included (17/39, 44% male). Of them, 44% (17/39) were allocated to the intervention group, and 56% (22/39) were allocated to the control group. The usability testing session involved 3 DM specialist nurses. All were female, 30-46 years old, and working in the DM field for 6-16 years.

#### Pilot Randomized Controlled Experiment

As the intervention and control groups were normally distributed, an independent samples *t* test was performed. There was no significant difference in the scores between the intervention group (mean 7.24, SD 2.46) and control group (mean 7.39, SD 2.56; t_37_=–0.19, *P*=.85; maximum score was 13). The frequency distribution of the scores for both groups is presented in Multimedia Appendix 12.

These results suggest that, for the given 2 case scenarios, no difference was observed in the accuracy of the decision made using the app compared to the use of paper-based guideline algorithms.

A score of 8 was determined as the minimum standard of safe care for the given 2 case scenarios. In the intervention group, 9 doctors (9/17, 53%) scored ≥8, whereas only 7 doctors (7/22, 32%) in the control group scored ≥8. However, when comparing their scores for each case individually, 10 doctors (10/17, 59%) and 17 doctors (17/22, 77%) from the intervention and control groups, respectively, scored a minimum of 3 in the first case scenario, while 10 doctors (10/17, 59%) and 11 doctors (11/22, 50%) from the intervention and control groups, respectively, got a minimum score of 5 in the second case scenario.

#### Satisfaction Questionnaire

All 17 questionnaires were completed, providing a 100% response rate (intervention arm). Nearly 50% (9/17) of the junior doctors indicated prior experience using a CDSS app. With regard to their overall impression, 83% (14/17) were satisfied or somewhat satisfied with the app. Ease and simplicity of the app were the most emphasized features: easy to use, user-friendly, straightforward, quick, simple flow, good presentation, intuitive user interface, clear design, easy to input information, gives a good recommendation based on results, not too wordy, good font size, and much easier to use than the algorithm. By contrast, the most common negative points were the ambiguity of the navigation between pages or recommendations and difficulty scrolling up and down. Regarding usability, some respondents reported that they encountered some technical bugs during the session.

In terms of suggestions for improvement, participants expressed the need for easy and clear navigation; information on doses supported with links to evidence; additional information on drug side effects, for example weight loss or gain; the possibility to save previous searches to go back to them easily; the ability to enter patient’s current medications to help streamline the options at the end; and more specific advice regarding which combinations of dual or triple therapy would be more appropriate.

All participants thought that the app seemed useful and were willing to use such apps in their clinical practice, specifically for more complex patients when they are uncertain and to avoid searching through guidance.

#### Usability Testing

The DM specialist nurses faced several usability problems. The ambiguity of the navigation between pages or recommendations as well as app crashes were described as the most frustrating part of the app. Additionally, some DM specialist nurses felt that some data items were irrelevant, for example, when the app asks for blood pressure while looking at glycemic control. By contrast, they asked to add some data items such as BMI.

Nurses indicated several positive aspects of the app. They found it very useful that they could click on the drug names for further information. The DM specialist nurses stated also that the app was a good idea, particularly from a practice point of view. Although usability issues were experienced with the app, they did not hinder completion of the tasks. Moreover, they liked having the button “how did I get here,” which allowed them to check if they have inputted something incorrectly at any point. They also suggested having this screen compulsory in order to enable users to make sure their information was correct.

Several further suggestions were given by the DM specialist nurses to improve the app; for instance, when the app recommended monitoring the patient, nurses wondered about how to monitor; they indicated a preference to use other gestures when communicating with the app such as swiping; and there were thoughts that providing background information (such as basic guidelines or treatment pathways) on the home page would be helpful.

## Discussion

### Principal Findings

The current feasibility study aimed to develop and evaluate the impact, usability, and acceptability of a mobile CDSS app from the perspectives of health care providers for patients with DM and CKD. DM specialist nurses were found not to be currently using apps in their clinical practice, while most nurses were not even aware of existing medical apps. However, they appreciated the potential benefits that apps may bring to their clinical practice. Taking into consideration the needs and preferences of end users, a new mobile CDSS app, “Diabetes & CKD,” was developed. The evaluation of this app showed that there was no significant difference between using the app and the paper-based version of the app’s algorithm. Furthermore, the results from the satisfaction questionnaires found that most participants were satisfied with the app.

### Limitations

The results of the present study should be interpreted taking into account potential strengths and limitations. As regards external validation, the generalizability of findings to the entire population of health care providers is limited due to the sampling method used in the interview study and absence of statistical power in the controlled experiment. Other groups of health care providers, such as male nurses or general practice doctors, or another setting might yield a different result. Another aspect to be considered is that blinding of the intervention group is impossible due to the use of the app. Furthermore, this was the first time all the participants had used the app. Therefore, a learning effect should be taken into account. Finally, a possible weakness of the experimental design is the inability to control, completely, for all other confounders that might influence the outcome.

### Comparison With Prior Work

Limited small-scale, quantitative, efficacy studies have evaluated the use of mobile apps in diabetes care, although studies have become more numerous since 2014. In these studies, apps were mainly developed for self-management and were evaluated by patients with DM and rarely by health care providers [19,29-31]. When these apps were evaluated by experts in the field of health care–related mobile apps, it seems that only 9 of 65 apps could be helpful for self-management of DM based on the included variables [32]. Regarding health care providers’ perspectives and intention to adopt mobile technology, they seem to be positive [33], with perceived benefits and value to mainly motivate physicians to use mobile diabetes monitoring [34]. Therefore, the development of more apps, based on guidelines and intended for use by health care providers, seems to be not only acceptable and desirable but also of major importance.

In a recently published study, a CDSS app was developed for patients with type 1 DM. The authors took into account the needs and perspectives of patients with type 1 DM as well as of their parents in order to develop an app that provides patient-doctor communication, a diabetes diary, diabetes education, peer support, blood glucose test reminder, and abnormal blood glucose reminder. However, this app was not further evaluated after the development. Also, although doctors or nurses will be called to use this app in order to see patients’ diaries or laboratory results, they were not interviewed either for their needs or for their final impression of the app [35]. An application for type 2 DM was developed in 2017, using 3 main steps: identification of end users’ needs and perspectives, development, and evaluation of the final app. Importantly, authors recruited both patients with DM and health care providers in order to provide a more holistic approach [36]. The findings of the current study are not comparable with these studies even though these studies are in the field of DM and health care providers will use them. None of the studies described have the same aim and objectives as the current study and, thus, have different findings. This is because they have different designs, settings, and reported outcomes. Although the aim and design of currently published studies are different, the general message remains in accordance with our results, that technology is well accepted from both patient and health care worker perspectives [37]. Additionally, appropriate utilization in clinical practice could provide great benefits in the management of patients with DM [38,39].

Following a review of available literature, only 1 publication was identified that explored similar aims to those in our study. Kart et al [40] published a protocol for an upcoming study aiming to develop a user-friendly CDSS for the screening, diagnosis, treatment, and monitoring of DM diseases for physicians and patients in primary care. The clinical result of the decisions made by the app will be evaluated following a 6-month usage period. However, the results of this study have not yet published.

### Conclusions

To our knowledge, this is the first study to design, develop, and evaluate a CDSS app for DM and CKD based on the principles of user-centered design for health care providers. The methodology chosen ensured a rigorous exploration of a complex intervention. The study design carefully considered the needs and preferences of end users in order to increase its acceptability and utilization. Moreover, the pilot randomized controlled trial was the first attempt to test a mobile diabetes CDSS app for health care providers in a controlled environment using case scenarios.

The outcome of this feasibility study will guide the design of future CDSS apps in the field of DM, aiming to help health care providers with a personalized approach depending on patients’ needs (such as comorbidities), but always in accordance with guidelines. As the current findings indicate a lack of apps for health care providers but also positive feedback and acceptance from providers, the development of CDSS in the field of DM seems crucial and requires further robust evaluation.

## Appendix

Multimedia Appendix 1 Ethics approval and ethical considerations for the first step.

Multimedia Appendix 2 Recruitment and interview process.

Multimedia Appendix 3 Requirements.

Multimedia Appendix 4 Guidelines used in the development of the management pathways.

Multimedia Appendix 5 Decision algorithms.

Multimedia Appendix 6 Dose adjustments in chronic kidney disease.

Multimedia Appendix 7 Details for the 3 methods used in the evaluation procedure.

Multimedia Appendix 8 Evaluation stage: simulation-based case scenarios.

Multimedia Appendix 9 Evaluation stage: satisfaction questionnaires.

Multimedia Appendix 10 Participants characteristics in the “requirements gathering” step.

Multimedia Appendix 11 Examples of diabetes specialist nurses’ answers.

Multimedia Appendix 12 Frequency of scores in the control and intervention groups from the pilot randomized controlled experiment.

## Abbreviations

CDSS: clinical decision support system
CKD: chronic kidney disease
DM: diabetes mellitus
NHS: National Health Service
