# Supplementary material for: Mobile Clinical Decision Support System for the Management of Diabetic Patients With Kidney Complications in UK Primary Care Settings: Mixed Methods Feasibility Study
Source: JMIR Diabetes. 2020 Nov 18;5(4):e19650. doi: 10.2196/19650 (PMC7710444; doi:10.2196/19650)
Supplement: Multimedia Appendix 1 [file diabetes_v5i4e19650_app1.docx]

**Multimedia Appendix 1.** Ethics approval and ethical consideration at the first step.

The NHS Trusts across West Midlands, which participated to the study, are: University Hospitals Coventry & Warwickshire NHS Trust, Sandwell & West Birmingham Hospitals NHS Trust, Walsall Healthcare NHS Trust, Shropshire Community Health NHS Trust, The Dudley Group NHS Foundation Trust, Heart of England NHS Foundation Trust and South Warwickshire NHS Foundation Trust.

Filled consent forms were collected from nurses who agreed to participate at the beginning of each interview. Nurses’ contact information was not collected unless they were willing to take part in the second phase of this research. To ensure the confidentiality of participant, each nurse was anonymised. Participants had the right to withdraw from the study freely at any time. Collected study information (including recorded interviews and consent forms) was only accessed by the research team. The study information was retained securely by the researcher; electronic copies of the interviews were stored on university-owned computers with access passwords, while paper records of the consent forms were stored in a locked cabinet in the office at the University of Warwick. Data will be retained for a period of 10 years in line with the University of Warwick’s policy on published data.
