# Supplementary material for: Mobile Clinical Decision Support System for the Management of Diabetic Patients With Kidney Complications in UK Primary Care Settings: Mixed Methods Feasibility Study
Source: JMIR Diabetes. 2020 Nov 18;5(4):e19650. doi: 10.2196/19650 (PMC7710444; doi:10.2196/19650)
Supplement: Multimedia Appendix 2 [file diabetes_v5i4e19650_app2.docx]

**Multimedia Appendix 2.** Recruitment and Interview process.

The recruitment procedure was performed mainly by e-mails. An e-mail was sent by the researcher to the Research & Development Department in each Trust asking for a list of emails for all DSNs. Next, invitation emails were sent to the provided mailing lists of DSNs. The interested DSNs were invited to contact the researcher. Furthermore, two diabetes and endocrinology consultants helped circulating the invitation to all DSNs in their department. DSNs who participated in the study were given a voucher as a reimbursement of their time, and were eligible to claim any possible travel expenses due to the study.

Face-to-face interview with diabetic specialty nurses was chosen to gain insight into individuals’ perceptions, viewpoints and challenges. Any possible misunderstandings between the interviewer and the participants could be identified and immediately addressed. All the interviews were semi-structured and guided by an interview schedule. Semi-structured interviews have a flexible structure with a fixed set of open-ended questions in order to allow the interviewee’s responses to direct the flow of the interview rather than the questions themselves, and to keep the discussions within the boundary of the research objective. This is a pragmatic choice, because semi-structured interviewing allows for depth of questioning with probing and detailed exploration of individual statements [1]. Additionally, one-to-one interviews fit better within the busy schedule of nurses.

Open questions were supported with a number of possible probes that could be explored further. At the beginning of each interview, a brief description of mobile apps and the concept of decision-support, along with some examples, were provided. Then, some demographic questions, such as age and gender, along with other relevant questions (e.g., medical profession, level of clinical experience in years, and smartphone/tablet ownership), were included. The discussion questions were:

- Can you please tell me about your experience of using mobile applications (these are sometimes referred to as “mobile apps” or “apps”)?

- In your opinion, how might mobile apps help diabetes professionals during clinical work?

- What are the potential benefits of using mobile apps at the point of care?

- What are the potential limitations of using mobile apps at the point of care?

- Could you describe any challenges/difficulty you are facing in managing your patients where mobile apps may support you?

- How likely would you be to use a mobile app? Why, why not?

- Thank you for all the information you have provided, is there anything else you’d like to add before we end?

**References**

1. Robson CM, K. . Real world research. 4th ed John Wiley & Sons 2016.
