# Supplementary material for: Mobile Clinical Decision Support System for the Management of Diabetic Patients With Kidney Complications in UK Primary Care Settings: Mixed Methods Feasibility Study
Source: JMIR Diabetes. 2020 Nov 18;5(4):e19650. doi: 10.2196/19650 (PMC7710444; doi:10.2196/19650)
Supplement: Multimedia Appendix 3 [file diabetes_v5i4e19650_app3.docx]

**Multimedia Appendix 3.** Requirements.

Functional requirements

It is a single function app and hence, there is only one key requirement. The app needed to allow the user to enter patient’s parameters and convert them into recommendations. In other words, the app generates patient-specific advice to help clinicians in making informed decisions.

Technical requirements

The main non-functional requirements are as follows:

▪ The app needed to work across all mobile platforms and with all smartphones and tablets running on all operating systems,

▪To be downloadable on devices,

▪ To support offline mode,

▪ The app may run within any evergreen browser,

▪ No patient’s identifiable data are needed in the app,

▪ No user account or login data are needed to use the app,

▪ The number of data entries into the app kept to the minimum, including only the data elements that impact on the management plan,

▪ Inputted data stored temporarily on the device’s memory and then wiped at the end of a session or upon reloading or exiting the app; they are not kept for future sessions.

Medical requirements

The app needed to incorporate clinical management guidelines followed in the UK and best practices based on clinical guidelines, and to be updated regularly to reflect any update on the guidelines. The content of the app further needed to be verified by a number of diabetes and endocrinology consultants in order to ensure the accuracy of the given advice.
