# Supplementary material for: Mobile Clinical Decision Support System for the Management of Diabetic Patients With Kidney Complications in UK Primary Care Settings: Mixed Methods Feasibility Study
Source: JMIR Diabetes. 2020 Nov 18;5(4):e19650. doi: 10.2196/19650 (PMC7710444; doi:10.2196/19650)
Supplement: Multimedia Appendix 4 [file diabetes_v5i4e19650_app4.docx]

**Multimedia Appendix 4.** The guidelines used in the development of the management pathways.

▪ NICE guidelines [NG28] Type 2 diabetes in adults: management, December 2015, Last updated 2019 [1].

▪ NICE guidelines [CG182] Chronic kidney disease in adults: assessment and management, July 2014, Last updated 2015 [2].

▪ NICE guidelines [CG127] Hypertension in adults: diagnosis and management, August 2011-replaced [3].

▪ NICE guidelines [CG181] Cardiovascular disease: risk assessment and reduction, including lipid modification, July 2014, Last updated 2016 [4].

▪ NICE technology appraisal guidance [TA288] Dapagliflozin in combination therapy for treating type 2 diabetes, June 2013, Last updated 2016 [5].

▪ NICE technology appraisal guidance [TA315] Canagliflozin in combination therapy for treating type 2 diabetes, June 2014 [6].

▪ NICE technology appraisal guidance [TA336] Empagliflozin in combination therapy for treating type 2 diabetes, March 2015 [7].

▪ Clinical Practice Guideline on management of patients with diabetes and chronic kidney disease stage 3b or higher (eGFR <45 mL/min) [8].

**References**

1. Excellence NIfHaC. Type 2 diabetes in adults: management. 2015.

2. Excellence NIfHaC. Chronic kidney disease in adults: assessment and management. 2014.

3. Excellence NIfHaC. Hypertension in adults: diagnosis and management. 2011.

4. Excellence NIfHaC. Cardiovascular disease: risk assessment and reduction, including lipid modification. . 2014.

5. Excellence NIfHaC. Dapagliflozin in combination therapy for treating type 2 diabetes. 2013.

6. Excellence NIfHaC. Canagliflozin in combination therapy for treating type 2 diabetes. 2014.

7. Excellence NIfHaC. Empagliflozin in combination therapy for treating type 2 diabetes. 2015.

8. Guideline development g. Clinical Practice Guideline on management of patients with diabetes and chronic kidney disease stage 3b or higher (eGFR <45 mL/min). *Nephrol Dial Transplant.* 2015;30 Suppl 2:ii1-ii142.
