# Supplementary material for: Mobile Clinical Decision Support System for the Management of Diabetic Patients With Kidney Complications in UK Primary Care Settings: Mixed Methods Feasibility Study
Source: JMIR Diabetes. 2020 Nov 18;5(4):e19650. doi: 10.2196/19650 (PMC7710444; doi:10.2196/19650)
Supplement: Multimedia Appendix 5 [file diabetes_v5i4e19650_app5.docx]

**Multimedia Appendix 5.** Decision algorithms.

**Algorithm for diabetes and CKD stage 1 – 2 (60 ≤ eGFR ≤ 90)**

ACR<30

**Algorithm for diabetes and CKD stage 3 – 5 (eGFR<60)**

ACR ≥ 100

Haematuria? AND NO microvascular disease?

No

Yes

(HbA1c>58 AND maximal oral anti-diabetic therapy) OR (BP>150/90 AND using >3 anti-hypertensive drugs) OR (6 ≤ K+<3.5 AND unexplained)

No

Yes

No

No

No

Refer to Diabetes Renal Clinic

Check eGFR and ACR annually AND Go to Glycaemic Control Algorithm OR Hypertension Management Algorithm according to their values

Yes

Yes

Yes

Refer to Urology

Refer to Nephrology

Haematuria? AND Age ≥ 45

Visible Haematuria? OR Symptoms of urinary tract obstruction? OR Accelerated progression of CKD?

Yes

No

Yes

Yes

Refer to Diabetes Renal Clinic

(HbA1c > 58 AND maximal oral anti-diabetic therapy) OR (BP > 150/90 AND using > 3 anti-hypertensive drugs) OR (6 ≤ K+<3.5 AND unexplained)

Check FBC yearly; iron deficiency anaemia is: ▪ diagnosed in stage 5 CKD with a ferritin <100 mcg/l ▪ considered in stage 3/4 CKD with a ferritin <100 mcg/l

eGFR<45

Refer to or discuss with Nephrology

Go to Glycaemic Control Algorithm OR Hypertension Management Algorithm according to their values

Haematuria AND No retinopathy or neuropathy?

Renal ultrasound

Yes

ACR ≥ 30

No

ACR<100

eGFR ≥ 30

Hypertension on 4 or more anti-hypertensive drugs? OR Sustained decrease in eGFR of >25% over 12 months and a change in CKD stage? OR Sustained decrease of eGFR >15 ml/min over 12 months? OR Renal anaemia (Hb <110 g/L, following exclusion of bleeding, deficiency states, or primary haematological problem)?

Yes

Yes

Yes

Yes

No

No

No

No

No

Repeat eGFR at 3/12; then check eGFR and ACR yearly

**Algorithm for glycaemic control**

**Initial therapy:**

No

No

No

No

No

No

Yes

Yes

Yes

Yes

Yes

Yes

Go to first intensification

Review the dose of Metformin

Stop Metformin

eGFR ≥ 30

Go to initial therapy – Metformin contraindicated or not tolerated

Modified-release Metformin tolerated?

Consider a trial of modified-release metformin

HbA1c ≤ target HbA1c

Standard-release Metformin tolerated?

Standard-release Metformin with caution for those at risk of a sudden deterioration in kidney function and those at risk of eGFR falling below 45. Gradually increase the dose of standard-release metformin over several weeks to minimise the risk of gastrointestinal side effects

eGFR ≥ 45

Metformin contraindicated or not tolerated?

?

HbA1c > target HbA1c on lifestyle intervention

**Initial therapy – Metformin contraindicated or not tolerated:**

^1^ Do not offer or continue **Pioglitazone** if the patient has any of the following:

- heart failure or history of heart failure
- hepatic impairment
- diabetic ketoacidosis
- current, or a history of bladder cancer
- uninvestigated macroscopic haematuria.

No

No

Yes

Go to first intensification

Yes

HbA1c ≤ target HbA1c

Drug treatment tolerated?

Initial drug treatment with:

- A DPP-4i, **or**
- Pioglitazone^1^, **or**
- Repaglinide, **or**
- An Sulfonylurea

Discuss the benefits and risks of the options available, and base the choice of drug treatment on: effectiveness in terms of metabolic response, safety and tolerability, individual clinical circumstances, individual preferences and needs, the licensed indications or combinations available, and cost

HbA1c > target HbA1c on lifestyle intervention

**First intensification:**

^1^ Interrupt treatment with the SGLT-2i in patients who are hospitalised for major surgery or acute serious illnesses, and treatment may be restarted once the patient’s condition has stabilised

^2^ No licensed combination containing Repaglinide that can be offered at first intensification

Yes

Yes

Yes

No

No

No

Go to second intensification

HbA1c ≤ target HbA1c

Drug treatments tolerated?

Dual therapy with:^2^

- DPP-4i and Pioglitazone, **or**
- DPP-4i and an Sulfonylurea, **or**
- Pioglitazone and an Sulfonylurea

Metformin contraindicated or not tolerated?

HbA1c > target HbA1c on initial therapy

Dual therapy with:

- Metformin and a DPP-4i, **or**
- Metformin and Pioglitazone, **or**
- Metformin and an Sulfonylurea, **or**
- Metformin and an SGLT-2i^1^; only if eGFR ≥ 60

**Second intensification:**

HbA1c > target HbA1c on first intensification

Yes

No

Yes

Yes

Yes

Yes

Yes

^1^ An SGLT-2i in combination with insulin with or without other antidiabetic drugs is an option

^2^ Interrupt treatment with the SGLT-2i in patients who are hospitalised for major surgery or acute serious illnesses, and treatment may be restarted once the patient’s condition has stabilised

^3^ Adjust accordingly for people from black, Asian and other minority ethnic groups

^4^ Liraglutide and Dulaglutide are licensed to be used only if eGFR ≥ 30, and all GLP-1 mimetic should be stopped if eGFR<30

No

No

No

No

No

Insulin detemir

HbA1c ≤ target HbA1c

Insulin glargine

Biphasic or other pre-mixed Insulin

Reduction of at least 11 mmol/mol (1.0%) in HbA1c **AND** weight loss of at least 3% of initial body weight in 6 months

NPH Insulin

(BMI ≥ 35^3^ **AND** specific psychological or medical problems associated with obesity) **OR** (BMI<35 **AND** (Insulin therapy have significant occupational implications **OR** weight loss considered beneficial))

Combination therapy with Metformin, a Sulfonylurea and a Glucagon-Like Peptide-1 (GLP-1) mimetic^4^

Is NPH appropriate for patient?

Triple therapy not effective, not tolerated, or contraindicated?

Metformin + start Insulin-based treatment

Triple therapy with:

- Metformin, an Sulfonylurea and a DPP-4i, **or**
- Metformin, Pioglitazone and an Sulfonylurea, **or**
- Metformin, Pioglitazone and an SGLT-2i^2^ (not Dapagliflozin); only if eGFR ≥ 60, **or**
- Metformin, an Sulfonylurea and an SGLT-2i^2^ (not Dapagliflozin); only if eGFR ≥ 60

Insulin-based treatment^1^

Metformin contraindicated or not tolerated?

**Algorithm for management of hypertension**

BP > target BP **OR** ACR ≥ 3 **OR** eGFR<60 **OR** CVD indications

**Step 1 treatment**

Yes

ACE inhibitor plus either a diuretic or a generic CCB

Ethnic group=Black African or Caribbean

Yes

No

No

Yes

Yes

Yes

Yes

Yes

No

No

No

No

No

Investigate other causes of deterioration in renal function such as volume depletion or concurrent medication e.g. NSAIDs

Stop the ACE or ARB, or reduce the dose to a previously tolerated lower dose and go to step 2 treatment if required

Do not modify the dose, and repeat the test in 1 - 2 weeks

Treat the other factor and retest

Other causes found

eGFR > baseline GFR – (25 * baseline GFR)/100 **OR** Serum Creatinine<baseline Serum Creatinine + (30 * baseline Serum Creatinine)/100

Stop ACE or ARB and other drugs known to promote hyperkalaemia

, and go to step 2 treatment

Serum potassium concentration ≥ 6

^1^ If concurrent prescription of drugs known to promote hyperkalaemia, more frequent monitoring of serum potassium concentration is needed

Measure serum potassium concentrations and estimate the GFR between 1 and 2 weeks^1^

A once-daily, generic ACE inhibitor, but for a person with continuing intolerance to an ACE inhibitor (other than renal deterioration or hyperkalaemia), substitute an ARB for the ACE inhibitor

Do not offer ACE or ARB, and go to step 2 treatment

Serum potassium concentration > 5

Investigate and treat other factors known to promote hyperkalaemia

Pre-treatment serum potassium concentration > 5

CCB

Pregnant or of child-bearing potential

Yes

**Step 2 & 3 & 4 treatment**

Yes

Yes

Yes

No

Seek expert advice

Monitor BP every 4-6 months

BP ≤ target BP

Add an alpha-blocker, a beta-blocker or a potassium-sparing diuretic (the last with caution if the individual is already taking an ACE inhibitor or an ARB)

BP ≤ target BP

Add the other drug (that is, the CCB or diuretic)

BP ≤ target BP

Add a diuretic (usually a thiazide or thiazide-related diuretic

CBB not tolerated **OR** not suitable because of oedema **OR** there is evidence of heart failure **OR** a high risk of heart failure

Add a CCB

BP > target BP on step 1 treatment

Yes

No

No

No
