# Supplementary material for: Mobile Clinical Decision Support System for the Management of Diabetic Patients With Kidney Complications in UK Primary Care Settings: Mixed Methods Feasibility Study
Source: JMIR Diabetes. 2020 Nov 18;5(4):e19650. doi: 10.2196/19650 (PMC7710444; doi:10.2196/19650)
Supplement: Multimedia Appendix 6 [file diabetes_v5i4e19650_app6.docx]

**Multimedia Appendix 6.** Dose adjustments in chronic kidney disease.

|  | **Drug** | **CKD-1**  **(GFR≥90)** | **CKD-2**  **(60≤GFR≤89)** | **CKD- 3a**  **(45≤GFR≤59)** | **CKD- 3b**  **(30≤GFR≤44)** | **CKD-4**  **(15≤GFR≤29)** | **CKD-5ND**  **(GFR<15)** | **CKD-5D**  **(GFR<15)** |
| --- | --- | --- | --- | --- | --- | --- | --- | --- |
| **Biguanides** | **Metformin** | **No adjustments** | | **1.5g-850 mg/day; do not initiate if eGFR <45** | | **To be avoided** | | |
| **Sulfonylureas** | **Glicazide** | **Start at low doses and dose titration every 1-4 weeks (High risk of hypoglycemia)** | | | | | | |
|  | **Glimepiride** | **Reduce dosage to 1 mg/day (High risk of hypoglycemia)** | | | | | | |
| **Meglitinides** | **Repaglinide** | **No adjustments** | | | | | **Limited experience available** | |
|  | **Nateglinide** | **No adjustments** | | | | | **Start at 60 mg/day** | **To be avoided** |
| **TZD’s** | **Pioglitazone** | **No adjustments** | | | | | | |
| **DPP-4 inhibitors** | **Sitagliptin** | **No adjustments** | | **Reduce to 50 mg/once daily if eGFR 30-50** | | **Reduce to 25 mg/once daily** | | |
|  | **Vildagliptin** | **No adjustments** | | **Reduce to 50 mg/once daily if eGFR <50** | | | | |
|  | **Saxagliptin** | **No adjustments** | | **Reduce to 2.5 mg/once daily** | | | | |
|  | **Linagliptin** | **No adjustments** | | | | | | |
|  | **Alogliptin** | **No adjustments** | | **Reduce to 12.5 mg/once daily** | | **Reduce to 6.5 mg/once daily; and use with caution** | | |
| **Incretin mimitics**  **(GLP-1)** | **Exenatide** | **No adjustments** | | **Careful use if eGFR 30-50** | | **To be avoided** | | |
|  | **Liraglutide** | **No adjustments** | | | | **To be avoided** | | |
|  | **Lixisenatide** | **No adjustments** | | **Careful use if eGFR 30-50** | | **To be avoided – no information available** | | |
|  | **Dulaglutide** | **No adjustments** | | | | **To be avoided** | | |
| **SGLT-2 inhibitors** | **Dapagliflozin** | **No adjustments** | | **To be avoided - ineffective** | | | | |
|  | **Canagliflozin** | **No adjustments** | | **Reduce to 100 mg/once daily** | **To be avoided** | | | |
|  | **Empagliflozin** | **No adjustments** | | **Reduce to 10 mg/once daily** | **To be avoided** | | | |

**CKD:** Chronic Kidney Disease, **eGFR:** estimated Glomerular Filtration Rate, **TZD:** Thiazolidinediones, **DDP-4:** Dipeptidyl Peptidase 4, **GLP-1:** Glucagon-like Peptide-1, **SGLT-2:** Sodium-glucose co-transporter-2
