# Supplementary material for: Mobile Clinical Decision Support System for the Management of Diabetic Patients With Kidney Complications in UK Primary Care Settings: Mixed Methods Feasibility Study
Source: JMIR Diabetes. 2020 Nov 18;5(4):e19650. doi: 10.2196/19650 (PMC7710444; doi:10.2196/19650)
Supplement: Multimedia Appendix 7 [file diabetes_v5i4e19650_app7.docx]

**Multimedia Appendix 7.** Details for the three methods used at the evaluation procedure.

Pilot randomised controlled experiment

A pilot randomised controlled experiment was carried out using case scenarios to investigate the feasibility and impact of introducing a clinical decision-support app. Three case scenarios were prepared by a diabetes and endocrinology consultant. Randomisation sequence was generated using the random number generator in Microsoft Excel Software. The evaluation of the app first design was conducted to assess how the app could support HCPs in terms of (a) workflow efficiency (measured by time to complete the tasks), and (b) adherence to clinical guidelines (measured by accuracy of decision made, compared to the use of paper-based guideline algorithms).

The controlled experiment tests the hypothesis that clinicians’ work will be greatly aided -shorter time to make a decision and more accurate decision - by an app intervention that can provide decision-support on the care of patients with diabetes and CKD.

Satisfaction questionnaire

All participants in the intervention group were invited at the end of the piloting session to provide qualitative feedback, using a questionnaire to assess their subjective satisfaction from working with the app. The questionnaire had six openended questions:

-Do you have any prior experience of using a mobile-based platform as a decision-support tool?

-Your overall satisfaction with the app.

-Positive and negative things about the app.

-Usability issues if any was encountered during the session.

-Features to change/add.

-Your willingness to use the app in the future and why. (When appropriate to use the app?, When and why not appropriate to use the app?, Wishes/ suggestions for improvements.)

-Any other thoughts about the app.

Usability testing

Usability testing session was undertaken to assess the app’s interface and functionality and users’ attitudes towards the app. Participants tested the app in controlled conditions, performing representative tasks using case scenarios. They were asked to follow the ‘think-aloud’ protocol which asks them to verbalise what they are doing while they are doing it, which slows participants down considerably, therefore, measuring the time was considered not feasible in this session.
