# Supplementary material for: Mobile Clinical Decision Support System for the Management of Diabetic Patients With Kidney Complications in UK Primary Care Settings: Mixed Methods Feasibility Study
Source: JMIR Diabetes. 2020 Nov 18;5(4):e19650. doi: 10.2196/19650 (PMC7710444; doi:10.2196/19650)
Supplement: Multimedia Appendix 8 [file diabetes_v5i4e19650_app8.docx]

**Multimedia Appendix 8.** Evaluation stage: simulation-based case scenarios.

**Case Scenario 1:**

**Mrs**. **Smith**

A white female, 65 years of age, who has had Type 2 Diabetes for approximately 8 years and Hypertension for 5 years. She also has Stage 2 Chronic Kidney Disease. She presents after a recent ophthalmic examination that showed diabetic retinopathy with poor glycemic control despite receiving Metformin (2000 mg/d) plus a SGLT-2 Inhibitor (Dapagliflozin 10 mg OD) for the last 12 months. On review of systems, she indicates no weight change. She has mild Dyslipidemia controlled with a Statin and Hypertension treated with an Angiotensin II Receptor Blocker (ARB).

**How will you approach managing her glycaemic control?**

| Measure | Result | Measure | Result |
| --- | --- | --- | --- |
| **HbA_1c_** | 9% or 75mmol/mol | **ACR** | 110 mg/mmol |
| **Blood pressure** | 120/70 mmHg | **Serum potassium** | 3 mmol/litre |
| **eGFR** | 64 mL/min/1.73 m2 | **BMI** | 29 kg/m2 |

**HbA1:** Haemoglobin A1c**, ACR:** Albumin to Creatinine Ratio**, eGFR:** estimated Glomerular Filtration Rate**, BMI:** Body Mass Index

**Case Scenario 2:**

**Mr. John**

A white male, 46 years of age, who is registered as a new patient at the general practice. His medical history included Type 2 Diabetes and Hypertension for the past 11 years. Previously he had been told that he had protein in his urine. He now presents at the clinic with peripheral neuropathy. The patient had evidence of both microvascular and macrovascular disease. He also had microscopic haematuria. His GFR had deteriorated by 10ml/min (26%) over the past year from G3b to G4. Renal ultrasound shows normal-size kidneys. He was referred for smoking cessation counselling and given advice on weight reduction. His current medications include Metformin 1 g BD, Gliclazide 160 mg BD and Sitagliptin 100 mg OD, a Thiazide plus an Angiotensin-Converting Enzyme Inhibitor. His blood lipids are well controlled on Atorvastatin 10 mg daily.

**Task: How will you approach his glycaemic control management?**

| Measure | Result | Measure | Result |
| --- | --- | --- | --- |
| **HbA_1c_** | 76 mmol/mol (9.1%) | **ACR** | 200 mg/mmol |
| **Blood pressure** | 140/80 mmHg | **Serum potassium** | 3.5 mmol/litre |
| **eGFR** | 28 mL/min/1.73 m2 | **BMI** | 28 kg/m2 |

**HbA1:** Haemoglobin A1c**, ACR:** Albumin to Creatinine Ratio**, eGFR:** estimated Glomerular Filtration Rate**, BMI:** Body Mass Index
