# Supplementary material for: Mobile Clinical Decision Support System for the Management of Diabetic Patients With Kidney Complications in UK Primary Care Settings: Mixed Methods Feasibility Study
Source: JMIR Diabetes. 2020 Nov 18;5(4):e19650. doi: 10.2196/19650 (PMC7710444; doi:10.2196/19650)
Supplement: Multimedia Appendix 9 [file diabetes_v5i4e19650_app9.docx]

**Multimedia Appendix 9.** Evaluation stage: satisfaction questionnaires.

1. Do you have any prior experience of using a mobile-based platform as a decision-support tool?
2. Your overall satisfaction with the app.
3. Positive and negative things about the app.
4. Usability issues if any was encountered during the session.
5. Features to change/add.
6. Your willingness to use the app in the future and why.
   - When appropriate to use the app?
   - When and why not appropriate to use the app?
   - Wishes/ suggestions for improvements.
7. Any other thoughts about the app.
