# Supplementary material for: Mobile Clinical Decision Support System for the Management of Diabetic Patients With Kidney Complications in UK Primary Care Settings: Mixed Methods Feasibility Study
Source: JMIR Diabetes. 2020 Nov 18;5(4):e19650. doi: 10.2196/19650 (PMC7710444; doi:10.2196/19650)
Supplement: Multimedia Appendix 10 [file diabetes_v5i4e19650_app10.docx]

**Multimedia Appendix 10.** Participants characteristics at the ‘requirements gathering’ step.

| **Factor** | **N (%)** |
| --- | --- |
| **Gender**  Male  Female | 0 (0)  15 (100) |
| **Age group**  20-29  30-39  40-49  50-59 | 1 (7)  3 (20)  6 (40)  5 (33) |
| **Experience in diabetes (years)**  2-5  6-10  11-15  16-18 | 4 (27)  5 (33)  5 (33)  1 (7) |
| **Profession**  Paediatric DSN^a^  DSN^a^  Diabetes Service Lead | 2 (13)  12 (80)  1 (7) |
| **Work setting**  Community-based  Hospital-based  Both | 2 (13)  4 (27)  9 (60) |
| **Prescribing qualification**  Yes  No | 11 (73)  4 (27) |
| **Device ownership**  Smartphone  Tablet | 14 (93)  15 (100) |
| **Trust devices**  Basic mobile phone only  Smartphone only  Tablet only  Smartphone and tablet  Nothing | 5 (33)  3 (20)  3 (20)  3 (20)  1 (7) |

^a^DSN: Diabetic Specialist Nurse
