# Supplementary material for: Mobile Clinical Decision Support System for the Management of Diabetic Patients With Kidney Complications in UK Primary Care Settings: Mixed Methods Feasibility Study
Source: JMIR Diabetes. 2020 Nov 18;5(4):e19650. doi: 10.2196/19650 (PMC7710444; doi:10.2196/19650)
Supplement: Multimedia Appendix 11 [file diabetes_v5i4e19650_app11.docx]

**Multimedia Appendix 11.** Examples of diabetic specialist nurses’ answers

Experience of using apps

Nearly all nurses had prior experience in using mobile apps but only for personal purposes. Example: ‘*I use quite a lot of apps for personal purposes…*’ (Nurse 1). On the contrary, nurses had limited experience in using apps for clinical purposes. Example: ‘*I am very much a novice, so in terms of my work life, I don’t use them at all, except for the BNF app on my own private iPhone*.’ (Nurse 8). Also, most nurses were only aware of apps that support patients in self-care and monitoring of their diabetes. Most nurses lack knowledge about available apps in the market that may support them in clinical work. Examples: ‘*I don't know if there's any app out there to use them*.’ (Nurse 3) or ‘*I wasn't aware that there was this sort of thing, and then when you mentioned about wanting to meet to discuss today, I just thought, oh ok, so I had a little look... I know that there are lots of patient ones, I wasn't aware that there were more out there for professionals… But I did have a little bit of a search to see, and I did come across some other ones, which I felt um I found quite useful, actually*.’ (Nurse 7). However, most nurses tend to recommend the use of trusted apps to their patients. Example: ‘*We recommend Carbs & Cals to our Type 1 for carbohydrate counting*.’ (Nurse 4)

Perceptions and views towards using apps in clinical setting

Nurses have clear ideas about several potential benefits of using apps in clinical practice. The commonly reported benefit was the convenience, time and accessibility of apps due to the mobility of these devices, in particular for nurses working in the community since part of their work involves going to patients’ homes and schools. Example: ‘*It's quick, it's accessible immediately, you know, because we are not always hospital-based, that would be a huge benefit… So, it would be a bit of a timesaver for us as well maybe*.’ (Nurse 2) Further reported advantaged were the no need of written paper and the improvement of patients’ care.

Limitations were the funding and Internet access, technical issues related to WiFi connection, network coverage and poor signal in the hospital, the cost and the small screen size of some devices made them inappropriate to be used by people with visual impairment. Examples: ‘*Sometimes it's hard to get a signal on some of the areas because there's blind spots*.’ (Nurse 4) or ‘*And if there’s a cost implication. Because if it’s freeware obviously it’s okay, but if they have to pay for it. That might be a bit of a turning off point*.’ (Nurse 12)

The main concerns were confidentiality and information governance around apps that involve storing or accessing patients’ data. Example: ‘*I would be concerned about accessing patient records, because of the confidentiality… if you put patient's information on there, it would be, that would be the main concern, because you are out and about with it*.’ (Nurse 14)

Further concerns regard mainly reliable source, validation, safety, difficulty in use during consultation as it disables eye contact and outdated information.

Difficulties in diabetes management that might be supported by the use of apps

The main difficulty at this point was that some nurses were unable to envisage how apps could support their clinical decision-making process, although nurses were given a brief description of decision-support tools with examples at the beginning of each interview. Example: ‘*Oh do you mean people can't work it out for themselves; is it going to prompt you to make a decision? I don't think so really… Well, nothing can teach you to look after patients, you need that exposure and you need that experience, that clinical experience… And if you're always relying on technology, where's your experience, where's your clinical skill going to go?’* (Nurse 5)

As regards further equipment, examples: ‘… *when it comes to the app rather than having a lot of information on one app it would probably be split into certain sections so you might have an app which tells you section about drugs to use for certain conditions and then another one which tells you about how to treat a hypo and stuff like that. So you don’t have a big app which tells you a lot of information. But just small… Quick and easy to get to*.’ (Nurse 12) or ‘*If it is a clinical app, it has to follow local or national guidelines in respect to the information that it is giving*.’ (Nurse 15)

Willingness to use apps at the point of care

Overall, nurses expressed strong willingness to use apps in clinical practice. However, willingness was very much dependent on the benefits they get, and the simplicity of the app. Examples: ‘*Yeah, very likely. As long as it was user friendly…*’ (Nurse 2) or ‘*As long as it's just simple, simple information, I think that people are more likely to use it*.’ (Nurse 7) or ‘*Um just ease of use bearing in mind the time restrictions.*’ (Nurse 11) or ‘*Just make it really simple, mobile app for dummies*.’ (Nurse 14)
