# Supplementary material for: Mobile Clinical Decision Support System for the Management of Diabetic Patients With Kidney Complications in UK Primary Care Settings: Mixed Methods Feasibility Study
Source: JMIR Diabetes. 2020 Nov 18;5(4):e19650. doi: 10.2196/19650 (PMC7710444; doi:10.2196/19650)
Supplement: Multimedia Appendix 12 [file diabetes_v5i4e19650_app12.docx]

**Multimedia Appendix 12.** Frequency of scores at the control and intervention group from the pilot randomised controlled experiment.

**
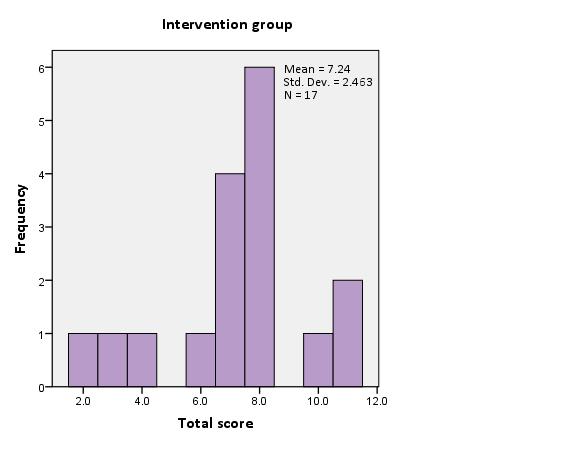
**

**
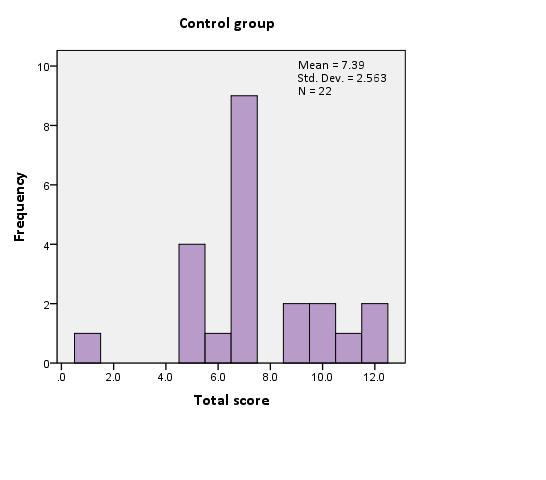
**
